# Low-Dose Pulsatile Interleukin-6 As a Treatment Option for Diabetic Peripheral Neuropathy

**DOI:** 10.3389/fendo.2017.00089

**Published:** 2017-05-02

**Authors:** April Ann Cox, Yves Sagot, Gael Hedou, Christina Grek, Travis Wilkes, Aaron I. Vinik, Gautam Ghatnekar

**Affiliations:** ^1^FirstString Research, Mt. Pleasant, SC, USA; ^2^Relief Therapeutics SA, Zurich, Switzerland; ^3^Roper St. Francis Healthcare, Mt. Pleasant, SC, USA; ^4^Eastern Virginia Medical School, Norfolk, VA, USA

**Keywords:** diabetic peripheral neuropathy, interleukin-6, nerve regeneration, myokine, neurocytokine

## Abstract

Diabetic peripheral neuropathy (DPN) remains one of the most common and serious complications of diabetes. Currently, pharmacological agents are limited to treating the pain associated with DPN, and do not address the underlying pathological mechanisms driving nerve damage, thus leaving a significant unmet medical need. Interestingly, research conducted using exercise as a treatment for DPN has revealed interleukin-6 (IL-6) signaling to be associated with many positive benefits such as enhanced blood flow and lipid metabolism, decreased chronic inflammation, and peripheral nerve fiber regeneration. IL-6, once known solely as a pro-inflammatory cytokine, is now understood to signal as a multifunctional cytokine, capable of eliciting both pro- and anti-inflammatory responses in a context-dependent fashion. IL-6 released from muscle in response to exercise signals as a myokine and as such has a unique kinetic profile, whereby levels are transiently elevated up to 100-fold and return to baseline levels within 4 h. Importantly, this kinetic profile is in stark contrast to long-term IL-6 elevation that is associated with pro-inflammatory states. Given exercise induces IL-6 myokine signaling, and exercise has been shown to elicit numerous beneficial effects for the treatment of DPN, a causal link has been suggested. Here, we discuss both the clinical and preclinical literature related to the application of IL-6 as a treatment strategy for DPN. In addition, we discuss how IL-6 may directly modulate Schwann and nerve cells to explore a mechanistic understanding of how this treatment elicits a neuroprotective and/or regenerative response. Collectively, studies suggest that IL-6, when administered in a low-dose pulsatile strategy to mimic the body’s natural response to exercise, may prove to be an effective treatment for the protection and/or restoration of peripheral nerve function in DPN. This review highlights the studies supporting this assertion and provides rationale for continued investigation of IL-6 for the treatment of DPN.

## Background: Diabetic Peripheral Neuropathy (DPN)

A growing diabetes pandemic is unfolding not only in the United States, but also globally. According to the American Diabetes Association (ADA), the prevalence of diabetes in 2012 was 9.3%, with 86 million people in prediabetes staging. The cost of diabetes, in the US alone was reported to be 245 billion dollars in 2012, and diabetes is ranked the seventh leading cause of death. DPN is the most common complication associated with type 1 or type 2 diabetes (T1D, T2D), and has an expected lifetime prevalence of 50% of individuals suffering with diabetes (1, 2). It is noteworthy, that outside of blood glucose management, drug treatment strategies for DPN are currently limited to analgesic agents targeting the neuropathic pain associated with DPN (3). Besides their partial efficacy on pain, these treatments do not address the non-painful symptoms of the disease nor halt worsening of symptoms. DPN is the primary risk factor associated with foot ulceration, amputation, falls, fractures, and traumatic brain injury; these serious complications highlight the importance of finding disease modifying treatment strategies to retard DPN progression and/or severity (4).

Diabetic peripheral neuropathy as described by the Toronto Consensus Panel on Neuropathies is “a symmetrical, length-dependent sensorimotor polyneuropathy attributable to metabolic and microvessel alterations as a result of chronic hyperglycemia exposure (diabetes) and cardiovascular risk covariates” (5). Symptomatology for DPN includes pain, burning, itching, tingling, and numbness displaying predominately a “glove and stocking” (hands and feet) distribution. Intraepidermal nerve fibers (IENF), those fibers found in the epidermis, are the nerves most associated with the symptoms of DPN. Sensory fibers, rather than motor, that conduct both pain and mechanical sensory inputs are the primary fiber type affected in DPN (6, 7). The etiology of sensory nerve dysfunction and degeneration in DPN, while inextricably linked to extended hyperglycemia, insulin deficiency, and dyslipidemia are not yet fully understood (5, 8). Additional associated co-factors such as oxidative stress, mitochondrial dysfunction, advanced glycation end products, activated protein kinase C, polyol pathway activation, and decreased neurotrophin production have also been identified as playing potential causative roles in the development and/or progression of DPN (9, 10). Some of the known anatomical changes to nerve fibers that occur in the DPN setting include axonal degeneration, Schwann cell loss, focal demyelination, decreased IENF density, and blood vessel loss (11). These pathological changes result in nerve dysfunction including decreased nerve conduction velocity and endoneurial perfusion resulting in DPN symptomatology. Given the multifactorial nature of the underlying pathophysiologies driving development of DPN, it follows that an ideal disease modifying treatment should be multimodal in nature to best restore nerve function.

## Interleukin-6 (IL-6), Exercise, and DPN: Making the Connection

There are no Food and Drug Administration (FDA)-approved therapeutics for the regeneration and/or repair of peripheral nerves in DPN, currently the recommended treatments are limited to diet and exercise (12). Of note, ongoing studies using the anti-convulsant topiramate are showing promising results for nerve regeneration as well as metabolic improvements. The ADA has added it to their recommended treatment regimen; however, additional studies will be required to garner FDA approval for the indication (13–15). Controlling glucose in the T1D population was shown to reduce the development of DPN by 64% (16). The potential of this strategy for treating DPN in the T2D population remains to be confirmed (1, 17, 18). Therefore, glycemic control alone, when attainable, does not confer complete protection, leaving a susceptible population (T2D) that requires additional treatment strategies.

By its nature, exercise provides multifactorial benefits in the prevention and treatment of DPN through stimulating blood flow and insulin response, modifying lipid metabolism, reducing chronic inflammation (19, 20), and more importantly stimulating IENF regeneration (21, 22). Exercise stimulates secretion of neurotrophic factors such as brain-derived neurotrophic factor (BDNF) (23), and IL-6 (24). It is noteworthy that the beneficial effects of exercise on DPN (21, 22, 25) or in healthy persons have been partially attributed to the transient secretion of IL-6 that occurs rapidly during/post exercise. IL-6 was first identified in 1985 as a B-cell stimulatory factor and described as a pro-inflammatory cytokine integral in initiating the acute phase response of the immune system (26, 27). Research now supports IL-6 as a multifunctioning cytokine capable of eliciting both pro- and anti-inflammatory effects in a context-dependent fashion (28).

Observations that IL-6 is acutely released from muscle cells in response to exercise have led to its further characterization as a myokine (29). In this role, IL-6 stimulates glucose uptake and/or increases insulin sensitivity (30–34). Subsequent to strenuous exercise in humans, the plasma concentration of IL-6 can increase up to 128 fold (marathoners pre-race <1.0 pg/mL, ~80 pg/mL post-race), returning to near baseline within 4 h (35). IL-6 response to exercise is seemingly dependent on type, duration, and intensity of exercise performed, e.g., in 2 min of sprint cycling IL-6 levels peaked at 10 vs. 35 pg/mL after 2.5 h of treadmill running (36–38). It is important to emphasize that the robust and transient elevation of IL-6 levels due to exercise is distinct from the chronically elevated IL-6 levels associated with T2D (non-diabetic healthy controls ~1.5 pg/mL vs. T2D patients ~2–5 pg/mL) (39–42). As exercise produces both an anti-inflammatory response and a transient elevation of IL-6, the two phenomena have been causally linked (34). Similarly, as the beneficial role of exercise in the treatment of DPN is paralleled by a transient increase in IL-6 levels (21, 35), we have hypothesized that a connection exists. Therefore, we have conducted a review of literature to further investigate how IL-6 may play a beneficial role in DPN.

## Clinical Studies: IL-6 Administration to Mimic Exercise

Numerous clinical studies have been conducted to investigate the effects of acutely administering exogenous IL-6 in both non-diabetic healthy and diabetic subjects. This transient elevation of IL-6 has been reported to increase circulating levels of the anti-inflammatory cytokines IL-10, IL-1ra (IL-1 receptor antagonist), and cortisol that result in leukocytosis similar to that seen after exercise (43). Additionally, IL-6 administration attenuates endotoxin-induced TNF-alpha production, further supporting an anti-inflammatory role of IL-6 (44).

Acute administration of IL-6 was shown to stimulate fat metabolism vs. glucose metabolism in skeletal muscle in healthy subjects (45). However, in a study in T2D subjects, IL-6 administration had no effect on insulin-stimulated glucose metabolism (46). Nevertheless, in this study as well as in several other studies performed in healthy volunteers (34, 35, 43, 47, 48) or T2D patients (34, 47), IL-6 infusion induced a significant decrease in circulating insulin, concomitantly with increases in lipolysis, without impacting glycemia or glucose uptake or release. Emerging preclinical research shows that IL-6 signaling following exercise increases glucose-transporter 4 (GLUT-4) expression resulting in increased insulin sensitivity (49). Altogether, these data suggest that, in addition to its activities on lipolysis, transient IL-6 might decrease insulin secretion and improve glycemia but not necessarily through a direct effect on glucose uptake but rather potentially through indirect mechanisms secondary to increased insulin sensitivity. It is important to note that these referenced studies all utilized a dosing paradigm designed to mimic the transient, pulsatile, and moderate elevation of IL-6 blood concentration (10–100 pg/mL) that occurs during exercise. In contrast, low-level chronically elevated circulating IL-6 (2–3 pg/mL) has been reported to be associated with an increased risk of developing diabetes and is reviewed elsewhere (33, 50, 51).

Of interest, T2D patients exhibit lower levels of exercise-induced IL-6 as compared to healthy individuals, suggesting that exercise does not fully activate repair mechanisms in these patients (42). It is therefore likely that exercise in T2D patients might exhibit reduced beneficial effects in regards to inflammation, glucose metabolism, and nerve regeneration than in healthy subjects. As more work is being performed to unravel the complexities of IL-6 signaling in the pre-diabetic and diabetic states, this seeming paradox of displaying both beneficial (when dosed to mimic exercise-induced IL-6) and detrimental (chronically elevated) effects may be better understood. Preclinical research in rodent DPN models utilizing a dosing strategy to reproduce IL-6 kinetics seen during exercise is beginning to shed some light on this topic.

## Preclinical Studies: IL-6 Administration in Rodent Models of Diabetic Neuropathies

The most widely used model of diabetic neuropathy is the streptozotocin (STZ)-induced rat model that displays decreased nerve blood flow, decreased nerve conduction velocity, and axonal degeneration of both sensory and motor fibers (52, 53), all pathological hallmarks of DPN. In the first study using the STZ model to investigate IL-6 as a treatment investigators compared various treatment regimens [subcutaneous vs. intra-peritoneal; daily, three times in a week (TIW) or weekly injections] of 1, 10, and 30 µg/kg body weight of recombinant glycosylated human IL-6 in young rats. For all regimens, IL-6 treatment dose-dependently improved muscle action potential (motor function), sensory nerve conduction velocity, IENF density, nerve fiber morphology (myelin thickness of sciatic nerve), and tail-flick latency. Shortly after, another group published similar findings, whereby IL-6 treatment improved DPN in an adult rat STZ model. Subcutaneous injection of IL-6 (1, 3, and 10 µg/kg TIW) improved several measures of nerve dysfunction including sensory and motor nerve conduction velocity, thermal hyperalgesia, tactile allodynia measures, and sciatic nerve endoneurial blood perfusion in a dose-dependent manner (54). This study reported an additional mechanism—vasodilation of the vasa nervorum, through which IL-6 may be signaling to restore nerve function.

The discovery that acute low-dose subcutaneous administration (1, 3, and 10 µg/kg TIW) of exogenous IL-6-induced improvements in nerve conduction and endoneurial perfusion were mechanistically expanded upon in subsequent studies (55). To uncover the mechanism through which IL-6 was signaling to trigger vasodilation, authors investigated the nitric oxide (NO) system in autonomic and vascular regulation as potential mechanistic targets of IL-6. Authors reported that IL-6 effects were not linked to NO signaling in either autonomic or vascular function, and hypothesized that improved neurovascular function may be mediated through the endothelium-derived hyperpolarizing (EDH) factor system (55). While there is no direct evidence to support this hypothesis, the authors offer support by citing a reference reporting that the cytokine leukemia inhibitory factor (LIF), closely related to IL-6, induced endothelium-mediated vasodilation (56). Knowing that 5′-AMP-activated protein kinase (AMPK) substantially mediates EDH response (57, 58) and that IL-6 stimulates AMPK activity (59–61), it might also be hypothesized that IL-6’s effects on microvessel dilation is mediated through AMPK activation.

In summary, to date, three preclinical studies have been conducted to evaluate the potential use of IL-6 as a treatment for DPN in rodent diabetic models (T2D). All three studies reported that IL-6 treatment resulted in improved or normalized nerve function and/or morphology. While none of these studies reported the corresponding plasma levels of IL-6 following administration, there are literature reports to establish normal rat plasma IL-6 levels to be 50–100 pg/mL (62–64), and after a single intraperitoneal injection of 25 µg/kg plasma IL-6 levels peaked at ~5,500 pg/mL dropping off to near baseline within 4 h (65). Taken together, the data demonstrate a transient 50- to 100-fold increase in IL-6 following a single dose administration may represent therapeutic range. It is important to note that elevated IL-6 levels in the STZ model associated with increased inflammation/pathological outcomes have also been reported (66–69). However, these reports are not directly investigating how exogenous pulsatile application of IL-6 affects diabetes symptoms, rather providing correlative findings, and as such are not discussed herein.

Interestingly, similar results were obtained with low-dose IL-6 treatment on chemotherapy-induced neuropathy models (70), highlighting that in addition to metabolic targets, IL-6 also exerts neuroprotective effects on nerve cells outside of the diabetic milieu. Other members of the IL-6 family of cytokines that share gp130 as a common signal transduction element, such as ciliary neurotrophic factor, LIF, and cardiotrophin-1 have also been studied as potential nerve protectant agents (71–76). While there is a growing body of support, the exact mechanisms of IL-6-induced improvements remain unknown. Given the ubiquitous nature of IL-6 expression, it seems likely that the underlying mechanisms driving protection and repair of peripheral nerves in DPN are multifactorial and act synergistically. To begin exploring some of these potential mechanisms, Schwann and nerve cell responses to IL-6 are presented.

## IL-6 and Schwann Cells: Stimulating Remyelination

Schwann cells, the myelin-producing cells of the peripheral nervous system, are integral to the healthy function of peripheral nerves. There is a growing body of literature investigating the role of IL-6 on Schwann cells and myelin expression. Expression studies in rat have shown IL-6 receptor alpha (IL-6Rα) expression in myelinating Schwann cells at the nodes of Ranvier, and in distinct membrane domains of the internodal cytoplasm (77). This expression pattern suggests that IL-6 may play a role in the maintenance between the myelinating Schwann cell and underlying nerve axon. Additionally, IL-6Rα expression is upregulated in response to sciatic nerve injury, in particular during the remyelination phase of injury, suggesting a role in the regenerative phase (77). One function of early secretion of IL-6 by denervated Schwann cells is to favor monocyte recruitment, probably via LIF regulation, to clear axon and myelin debris, a key prerequisite to successful regeneration (78). Indeed, given studies in STZ-induced diabetic models have shown that delayed Wallerian degeneration is related to impaired axonal regeneration (79, 80), it may be inferred that in the diabetic state a lack of sufficient IL-6 signaling in Schwann cells may serve to delay Wallerian degeneration, thus further impairing nerve regeneration. In cultured rat Schwann cells, IL-6 upregulates genes for abundant low molecular weight glycoproteins in myelin such as myelin basic protein (MBP), peripheral myelin protein P_0_ (81, 82) or peripheral myelin protein 22 (pmp22) via a JAK2-dependent pathway (83). Administration of IL-6/IL-6R fusion protein following nerve transection increased myelinated nerve fiber regrowth by fourfold (82).

*In vitro* models that support the approach of targeting human Schwann cell dysfunction in DPN have also been conducted. In a human Schwann cell culture model, BDNF treatment results in IL-6 secretion that is associated with JAK/STAT pathway activation and nerve regeneration (84). Additionally, it was reported that in hyperglycemic conditions, the expression of Na^+^ channel beta3 subunit in Schwann cells decreased, and treatment with IL-6 restored normal levels of beta3 subunit (85). In summary, Schwann cells, which display pathological microstructural changes in T2D may represent an integral target of IL-6 signaling (86, 87). In the DPN setting, application of exogenous IL-6 may promote remyelination of injured peripheral nerves by Schwann cells with subsequent improvements in nerve function.

## IL-6 and Nerve Cells: Stimulating Regeneration

Interleukin-6 is the founder cytokine of the neuropoietin family, is produced by both neurons and glia, and signals as a neurocytokine during both injury and regeneration states (88). IL-6 expression is generally restricted to traumatic conditions and provides temporary trophic support to induce repair response. A large body of evidence supports the role of gp130 cytokines (including IL-6) in preconditioning and triggering neuro-reparative responses (89). IL-6Rα and IL-6 signaling have also been linked to mediating chloride concentration rise in sensory neurons, a prerequisite to trigger nerve regeneration following injury, through phosphorylation of the cation-chloride cotransporter NKCC1 (90).

The ability of IL-6 to induce axonal regeneration has been demonstrated in many central nervous system paradigms even those highly refractory to neurite outgrowth (91, 92). IL-6, IL-6Rα, and gp130 mRNA are rapidly upregulated in peripheral nerves following injury (93–95). Studies using both *in vitro* and *in vivo* systems show that IL-6 can induce expression of growth-associated protein 43 (GAP-43), a protein involved in neurite pathfinding and neuronal network formation (96–98). IL-6 has been shown to act as a neurotrophin-enhancing cell viability and proliferation in a neuroblastoma cell line (99). In PC12 cells, IL-6/IL-6R fusion protein induced pituitary adenylate cyclase-activating polypeptide (PACAP), a strong inducer of neurite outgrowth (100). Surprisingly, PACAP produces cyclic AMP that, in turn, induces IL-6 transcription (101). One could imagine that such feedback mechanisms would lead to constitutively activated neurite outgrowth pathway; however, none of the studies using IL-6 transgenic mice report hyper-innervation. This positive retro-feedback is likely dampened by the negative feedback on IL-6 signaling mediated by SOCS3 (102).

Experiments using transgenic or knock-out (KO) mice confirmed the role of IL-6 in regulating nerve regeneration. Transgenic mouse models over-expressing human IL-6 and IL-6R show enhanced transection nerve regeneration (103). In a study where a preconditioning injury was performed on the sciatic nerve to stimulate IL-6-induced GAP-43 upregulation prior to injury, nerve regeneration following a subsequent crush injury was enhanced and the response blunted in IL-6 KO mice (97). IL-6 deficient mice display impaired sensory function (reduced amplitude of sensory action potentials and reduced temperature sensitivity) and impaired regeneration following sciatic crush injury as compared to wild-type controls (104), suggesting the important role of IL-6 in regeneration.

In summary, IL-6 signaling in an injury setting has been reported to induce nerve regeneration in numerous models, highlighting its neuro-regenerative properties. While diabetic neuropathies would not be considered as a traumatic pathology *per se*, a link of dysregulation of IL-6 production in nerve cells as a potential mediator of diabetic neuropathy has been suggested before (105). Altogether these data suggest a therapeutic opportunity for enhancing nerve regeneration with IL-6 treatment.

## Conclusion

**Figure 1:**
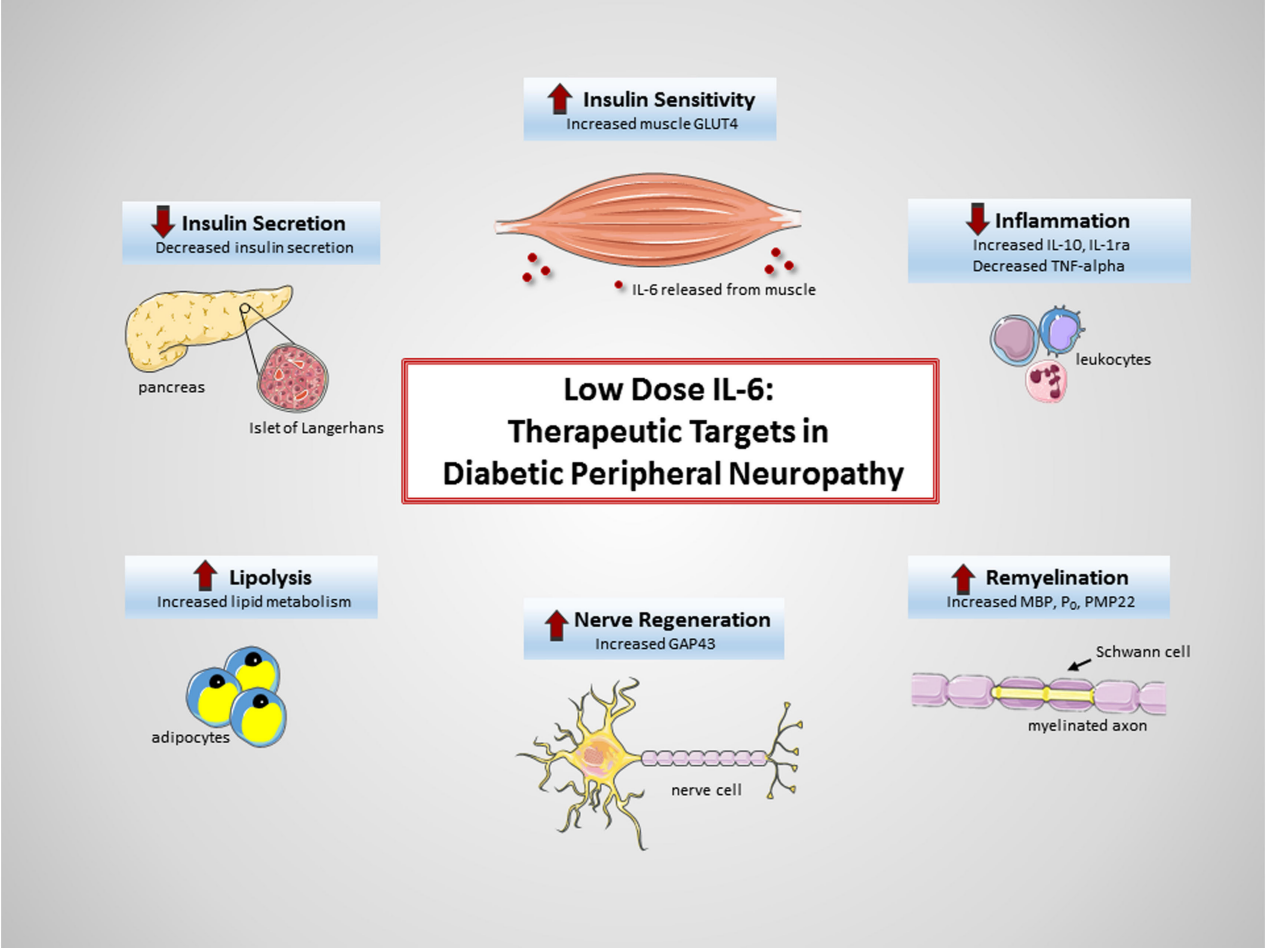
**Therapeutic targets of interleukin-6 (IL-6) in diabetic peripheral neuropathy (DPN)**. Exogenous administration of low-dose IL-6 to treat DPN  may be beneficial due to (1) increased insulin sensitivity in muscle (49), (2) decreased systemic inflammation (43, 44), (3) increased remyelination of axons (81–83), (4) increased nerve regeneration (96–98), (5) increased lipolysis (47, 48), and (6) decreased insulin secretion (34, 47).

Diabetic peripheral neuropathy remains one of the most common, serious, and potentially life-threatening complication of diabetes. Currently, there are no treatment options available to halt disease progression or restore nerve function in either DPN or autonomic neuropathy—a significant need remains. The multifunctional cytokine IL-6 has emerged as a potential therapeutic agent. While IL-6 is perhaps most classically known as a pro-inflammatory cytokine signaling in the immune system, research findings now support IL-6’s role as a myokine and neurocytokine capable of eliciting anti-inflammatory and regenerative responses. The acute exogenous administration of low-dose IL-6, dosed to mirror exercise-induced IL-6 kinetics, has shown significant protection and restoration of peripheral nerves in preclinical DPN models. A low-dose, pulsatile method of administering recombinant human IL-6 (Atexakin^®^ Alfa) is currently being developed, to continue investigating IL-6’s neuroprotective and regenerative capacity in the DPN setting. While the exact molecular mechanisms governing the effects are yet to be completely elucidated, it can be easily argued that IL-6 most likely elicits multiple beneficial effects across numerous cell and tissue types (Figure 1). This multi-targeted approach may prove to be highly efficacious in the complex setting of DPN.

## Author Note

Graphical abstract was generated using Servier Medical Art.

## Author Contributions

AC undertook the literature review, prepared the figure, and wrote the first draft of the manuscript. YS provided considerable scientific input and revision on early drafts of the manuscript as well as the final. All other authors, GH, CG, TW, AV, and GG, provided input and editing on the final draft of the manuscript.

## Conflict of Interest Statement

YS and GH are employed by Relief Therapeutics SA. Relief Therapeutics SA is currently developing Atexakin^®^ Alfa for diabetic peripheral neuropathy. AC, CG, and GG are employed by FirstString Research. FirstString Research has an ongoing collaboration with Relief Therapeutics SA for development of Atexakin^®^ Alfa in the United States. TW and AV declared no conflict of interest.
